# Detailed Molecular Mechanisms Involved in Drug-Induced Non-Alcoholic Fatty Liver Disease and Non-Alcoholic Steatohepatitis: An Update

**DOI:** 10.3390/biomedicines10010194

**Published:** 2022-01-17

**Authors:** Laura Giuseppina Di Pasqua, Marta Cagna, Clarissa Berardo, Mariapia Vairetti, Andrea Ferrigno

**Affiliations:** Unit of Cellular and Molecular Pharmacology and Toxicology, Department of Internal Medicine and Therapeutics, University of Pavia, 27100 Pavia, Italy; marta.cagna02@universitadipavia.it (M.C.); clarissa.berardo01@universitadipavia.it (C.B.); andrea.ferrigno@unipv.it (A.F.)

**Keywords:** drug-induced hepatic steatosis (DIHS), NAFLD, NASH, mitochondrial dysfunction

## Abstract

Non-alcoholic fatty liver disease (NAFLD) and non-alcoholic steatohepatitis (NASH) are some of the biggest public health challenges due to their spread and increasing incidence around the world. NAFLD is characterized by intrahepatic lipid deposition, accompanied by dyslipidemia, hypertension, and insulin resistance, leading to more serious complications. Among the various causes, drug administration for the treatment of numerous kinds of diseases, such as antiarrhythmic and antihypertensive drugs, promotes the onset and progression of steatosis, causing drug-induced hepatic steatosis (DIHS). Here, we reviewed in detail the major classes of drugs that cause DIHS and the specific molecular mechanisms involved in these processes. Eight classes of drugs, among the most used for the treatment of common pathologies, were considered. The most diffused mechanism whereby drugs can induce NAFLD/NASH is interfering with mitochondrial activity, inhibiting fatty acid oxidation, but other pathways involved in lipid homeostasis are also affected. PubMed research was performed to obtain significant papers published up to November 2021. The key words included the class of drugs, or the specific compound, combined with steatosis, nonalcoholic steatohepatitis, fibrosis, fatty liver and hepatic lipid deposition. Additional information was found in the citations listed in other papers, when they were not displayed in the original search.

## 1. Introduction

Non-alcoholic fatty liver disease (NAFLD), which includes both non-alcoholic fatty liver (NAFL) or simple steatosis and its progression to non-alcoholic steatohepatitis (NASH), is considered the hepatic manifestation of a condition called “metabolic syndrome”, which comprises a wide spectrum of metabolic abnormalities, such as simple steatosis, dyslipidemia, hypertension, insulin resistance and diabetes [1,2]. However, NAFLD is not always associated with metabolic syndrome. Exome-wide association studies demonstrated that the genetic variants located in 148Met allele of PNPLA3 and 167Lys allele of TM6SF2 genes are strictly associated with increased hepatic lipid deposition, steatohepatitis, cirrhosis and hepatocellular carcinoma, reducing at the same time the triglyceride plasma levels, the LDL-cholesterol concentrations and the risk of coronary artery disease [3]. In particular, the 148Met variant inhibits the proteasome-dependent degradation of PNPLA3, which accumulates and causes a decreased mobilization of triglycerides from the liver and a reduction in blood triglycerides concentration [4]. As regards the 167Lys variant allele in TM6SF2 gene, instead, it has been proposed to be responsible for the accumulation of toxic cholesterol in the liver and adipose tissue, moving it away from the blood vessels and protecting from cardiovascular diseases, but causing liver and adipose dysfunction [5]. Recently, some hepatologists proposed to replace the term NAFLD with metabolic associated fatty liver disease (MAFLD); however, at the moment, the nomenclature is still unchanged because the molecular bases and implications of the name switch are not fully understood; in addition, a premature change might create confusion [6], mainly because many clinical trials are currently specifically targeting NASH, which is not a major aspect in the concept of MAFLD. About a quarter of the world population suffers from NAFLD [7], characterized by intrahepatic lipid accumulation, with rates exceeding 43% in patients with metabolic syndrome [8]. Fortunately, only about 10% of patients suffering from NAFLD progress to NASH, even if this percentage is much higher in patients suffering from diabetes (37.7%), who present at the same time the highest prevalence rate of NAFL (55.5%), among the most important accompanying diseases [9]. In NASH, the hepatic fat deposition is accompanied by inflammation and increased free fatty acid oxidation, which can lead to a high risk of fibrosis, cirrhosis, and hepatocellular carcinoma development [10,11].

Among the various causes inducing steatosis and steatohepatitis, it has been estimated that a small percentage of cases, around 2%, are related to drug administration [12]. Drug-induced liver injury (DILI) associated with the onset and progression of NAFLD/NASH is known as drug-induced hepatic steatosis (DIHS). Many drugs are able to produce this side effect, including: antiarrhythmic drugs, antihypertensive drugs, anti-epileptic drugs and antineoplastic drugs. Generally, DIHS occurs in individuals who are genetically predisposed to develop steatosis, or who exhibit primary risk factors or comorbidities. DIHS has been described as a chronic disorder associated with therapies prolonged by weeks or months, but reversible after dismissing the treatment when it is possible [13]. In some cases, as in patients affected by acquired immune deficiency syndrome (AIDS) and subjected to highly active antiretroviral therapy (HAART) such as zidovudine, liver abnormalities are established and they may lead to death because of severe lipid dysregulation and accumulation, steatohepatitis, and acidosis [14,15,16,17]. Normally, livers already affected by steatosis are more prone to developing DIHS and, if this condition is concomitant with low hepatic energy production, the drugs can easily interfere with mitochondrial function, leading to further hepatic damage [13]. For this reason, many authors distinguish drugs able to induce steatosis and steatohepatitis into three categories: drugs that precipitate latent or already existing NASH as tamoxifen; drugs that can cause steatosis and steatohepatitis per se, such as the antiarrhythmic drugs amiodarone and perhexiline; drugs that induce sporadic events of NAFLD/NASH as carbamazepine [18]. The major mechanisms whereby drugs can induce NAFLD/NASH, as already mentioned, are by interfering with mitochondrial activity, inhibiting fatty acid oxidation, oxidative phosphorylation, and mitochondrial respiration [18]. However, NAFLD can be induced not only by mitochondrial dysfunction and inhibition of fatty acid oxidation, but also by dysregulation of lipid hepatic homeostasis in terms of fatty acid uptake, de novo lipogenesis (DNL), and transport by very low density lipoprotein (VLDLs) [19]. Thus, to better understand DIHS, it would be useful to briefly summarize the molecular mechanisms involved in the onset of hepatic steatosis, because many drugs can affect in different ways all of these processes. The aim of this work was precisely to provide a detailed picture of all the molecular mechanisms involved in drug-induced steatosis for eight different classes of compounds.

## 2. Molecular Mechanisms Involved in NAFLD Onset

Usually, an increase in hepatic lipid uptake and DNL occurs with a concomitant compensatory increment of fatty acid oxidation (FAO)) that, sometimes, is not sufficient to restore the normal content of lipids resulting in lipid accumulation. The consequent event is the initiation of hepatic damage and NAFLD progression triggered by oxidative stress, which leads to mitochondrial dysfunction and peroxisomal and cytochromes oxidation. Moreover, initially, the lipid export increases counteracting the fatty acid accumulation, but, at a certain point, this process reaches a plateau or even decreases during the disease progression, further promoting lipid accumulation [20]. 

A dysregulation of fatty acid uptake and in particular of the expression of specific transporters, such as: fatty acid transport protein (FATP), cluster of differentiation 36 (CD36), and caveolins, represents the first step of hepatic lipid accumulation. Knockdown and knockout mice for FATP2 and FATP5 showed a decreased fatty acids uptake and reduced hepatic steatosis after high-fat diet administration [21,22]; similarly, deletion of CD36 gene in mouse livers produced the same result [23]. Patients diagnosed with NAFLD or NASH, in fact, showed increased gene and protein expression of CD36, when compared with healthy controls [24], demonstrating the important role of this transporter in lipid accumulation. 

The liver can also synthesize lipids itself through DNL starting from acetyl-CoA, which is converted into malonyl-CoA by acetyl-CoA carboxylase (ACC). Malonyl-CoA becomes the substrate of fatty acid synthase (FAS) to produce palmitate. Many enzymes, such as desaturases [1] and elongases, rearrange the new fatty acid before its final esterification into triglyceride or exportation as VLDL. This process is finely regulated by two transcription factors: sterol regulatory element-binding protein 1c (SREBP-1c) and carbohydrate regulatory element-binding protein (ChREBP); SREBP-1c is activated by increased insulin and lipids, while ChREBP is regulated by increased carbohydrates [20]. NAFLD patients display abnormally elevated DNL when compared with healthy individuals [25]; in obese patients with NAFLD, about 26% of hepatic triglycerides come from DNL. In addition, in these patients, the regulation of DNL is lost when they pass from a fasting to a fed state [26].

The fate of fatty acids entering the hepatic cell is to form triglyceride stores, or to be oxidized to obtain energy in the form of ATP, in particular when circulating glucose levels are low. Oxidation of fatty acids occurs mainly in mitochondria, where the carnitine palmitoyltransferase 1 (CPT1) allows their entry, but it can also occur in peroxisomes and cytochromes, by β- and ω-oxidation, respectively. Regardless of where it occurs, FAO is regulated by peroxisome proliferator-activated receptor (PPAR)-α transcription factor activity [27,28]. PPARα activity promotes the transcription of genes related to mitochondrial, peroxisomal and cytochrome-mediated FAO as medium- and long-chain acyl-CoA dehydrogenases, acyl-CoA oxidase (ACOX) 1 and enoyl-CoA hydratase, CYP4A1 and CYP4A3, respectively, as demonstrated in PPARα deficient ob/ob mice [29]. In NASH patients, PPARα is downregulated and its expression decreases concomitantly with the increase in NAFLD activity score and fibrosis grade [30]. However, enhanced FAO produces a considerable quantity of ROS, which promotes inflammation and so the progression of the disease [31]. In this situation, a vicious circle is established: lipid oxidation and consequently ROS production induces mitochondrial DNA damage, further decreasing the mitochondrial activity and leading to their dysfunction [28], as reported in NASH overweight/obese patients [32,33].

**Figure 1:**
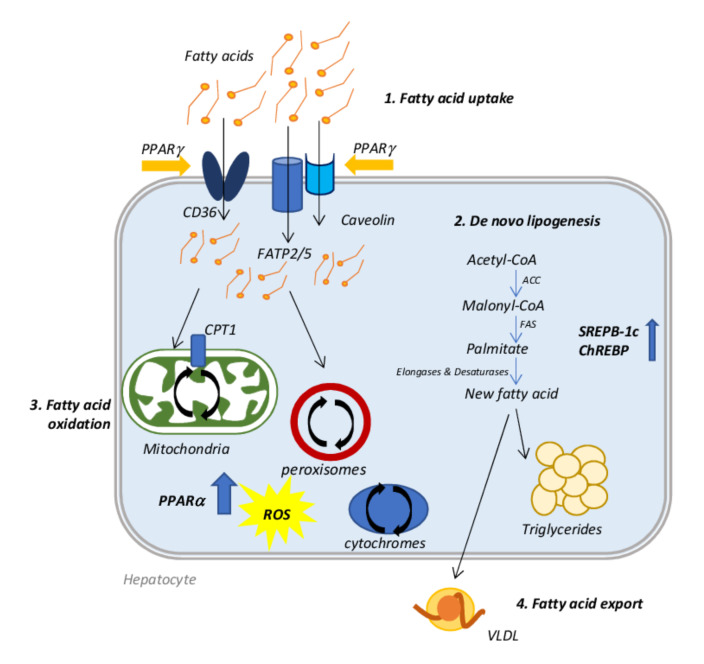
Schematic representation of the molecular mechanisms involved in NAFLD onset. 1. Fatty acid uptake: fatty acids are introduced in the cell by specific transporters such as CD36, FATP2/5 and caveolins, which are controlled by PPARγ transcriptional activity. 2. De novo lipogenesis: the liver synthesizes fatty acids starting from acetyl CoA. This mechanism is controlled by ChREBP and SREBP-1c activity. The new fatty acid can be stored as triglycerides or exported via VLDL formation. 3. Fatty acid export: VLDL particles are produced by lipidation of apoB100 in the ER and then they are transferred to the Golgi apparatus for a second lipidation that is necessary for maturation and export. 4. Fatty acid oxidation: Fatty acids introduced from the external environment or produced by de novo lipogenesis can be oxidized to form energy by mitochondrial and peroxisomal β-oxidation and by cytochrome ω-oxidation. All these processes produce ROS.

Lastly, lipid export is also affected in the setting of NAFLD. In the endoplasmic reticulum (ER), VLDL particles are produced by lipidation of apolipoprotein B100 (apoB100), a process catalyzed by the microsomal triglyceride transfer protein (MTTP). The product is transferred to the Golgi apparatus, where a second lipidation is required for the maturation of the VLDL particle [34]. In NASH patients, levels of apoB100 and MTTP may be diminished leading to a limited VLDL export, further exacerbating lipid accumulation [20] (Figure 1).

## 3. Drugs Inducing Hepatic Steatosis

The major drugs involved in steatosis onset are listed in the table below (Table 1).

### 3.1. Antiarrhythmic Drugs

Among the drugs listed in the category of antiarrhythmic agents, it is known that amiodarone and perhexiline can induce hepatic steatosis in a similar way.

Amiodarone belongs to the class III antiarrhythmic drugs; it is a complex compound that shows multiple electrophysiologic properties, unusual pharmacokinetics and various risky interactions and side effects. It has only been approved to treat life-threatening ventricular arrhythmias, but actually it is also used to treat atrial fibrillation [35]. Amiodarone is an iodine-containing compound similar to thyroxine and it is stored at high concentration in muscles, liver, lung and skin. It is lipid soluble and, probably, its prolonged half-life could be due to its slow release from tissues rich in lipids [36].

Perhexiline is an antianginal agent with few effects on blood pressure and heart rate, compared with other antianginal compounds [37]. It has a controversial history: because of a small number of cases of severe hepatotoxicity and neurotoxicity it was removed from the market in 1988, currently prescribed only in Australia and New Zeeland when no other option is available [38].

Both these drugs can induce hepatic lesions with a similar mechanism. In fact, they are both lipophilic compounds when they are in neutral form, but in their structure is present a secondary or tertiary amine that can be protonated, transforming these drugs into amphiphilic cations [39]. Prolonged and repeated administration of amiodarone and perhexiline can induce symptomatic liver disease in a small percentage (1–3%) of patients, which develop both micro- and macrosteatosis [40], with histopathological features resembling the alcohol-induced liver injury [40,41]. In particular, hepatocyte ballooning, Mallory Denk bodies and fibrosis are observed, accompanied by nuclear disorders, acidophilic bodies, foamy cells, glycogenated nuclei and portal hypertension. Moreover, after acute amiodarone administration, intracytoplasmic lamellar bodies were found in the hepatocytes, representing phospholipidosis [42,43]; the same lesions are typical of perhexiline administration [39]. Many mechanisms are involved in amiodarone and perhexiline induced microsteatosis and in particular, it has been demonstrated that they can inhibit both oxidative phosphorylation (OXPHOS) and mitochondrial fatty acid β-oxidation (FAO) [44]. When amiodarone or perhexiline, due to their amphiphilic properties, enter the intermembrane space of mitochondria, they undergo a protonation, generating cationic derivatives that freely enters the mitochondrion thanks to a favorable transmembrane potential [39,40]. This event has two consequences: these drugs rapidly and transiently uncouple the OXPHOS and, at the same time, the accumulation of cationic derivatives in the mitochondrial matrix causes the inhibition of different enzymes of the mitochondrial respiratory chain (MCR) and of FAO [44,45]. Whereas low concentrations of amiodarone and perhexiline can induce direct inhibition of FAO enzymes as CPT1 [44], higher concentrations are needed to impair MRC activity at the level of complexes I and II, as demonstrated by Fromenty et al. in 1990, using isolated liver mitochondria from mice exposed to 200 μM of amiodarone. The authors suggested that hydrophobic interactions of amiodarone with proteins and/or phospholipids of the inner membrane might be involved in the onset of these damages [46]. More recently, Spaniol and colleagues demonstrated on isolated rat liver mitochondria that amiodarone toxicity is related to the benzofuran structure and that also the complex III of the respiratory chain is affected [47]. It has been proposed that a moderate impairment of MRC activity could be involved in the transition from steatosis to steatohepatitis by means of an increase in ROS generation from complexes I and III [48]. ROS overproduction triggers lipid peroxidation and many other deleterious effects on the liver [44,49]. Moreover, it has been demonstrated that patients with specific CYP2D6 polymorphisms are at greater risk of developing steatohepatitis when administered with perhexiline, since CYP2D6 is the major enzyme in perhexiline metabolism [50].

**Figure 2:**
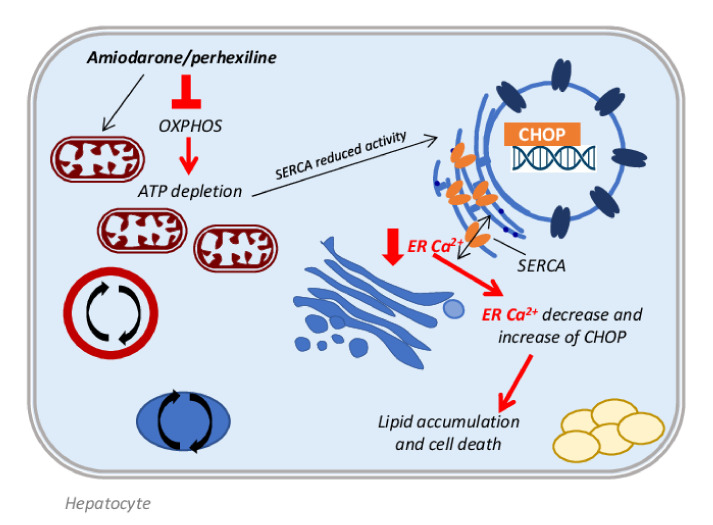
Schematic representation of one of the molecular mechanisms involved in amiodarone/perhexiline-induced NAFLD. The ATP depletion caused by amiodarone/perhexiline interference with OXPHOS leads to the reduced activity of the smooth endoplasmic reticulum Ca^2+^ pump (SERCA). The reduction in ER Ca^2+^ produces ER stress and the upregulation of CHOP activity with consequent increase in the activity of the lipid droplet proteins cell death activator (Cidea), cell death inducing DFFA like effector C (Cidec), and perilipin-2, which are involved in lipid accumulation.

A recent study by Erez et al. demonstrated in immortalized hepatocytes and in mice, that amiodarone administration strongly increases the expression of the spliced X-box binding protein 1 (sXBP1) and of its downstream target proteins, in particular CCAAT-enhancer-binding protein homologous protein (CHOP), markers of the endoplasmic reticulum (ER) stress response [51]. A possible explanation of the ER stress induction could be referred to the decrease in Ca^2+^ levels in the ER compartment, due to the amiodarone inhibition of mitochondrial respiration and to the ATP depletion, causing the reduced activity of the smooth endoplasmic reticulum Ca^2+^ pump (SERCA). This event induces the CHOP activation, which contributes to lipid accumulation by activating the lipid droplet proteins cell death activator (Cidea), cell death inducing DFFA like effector C (Cidec), and perilipin-2 (Figure 2). These data are supported by studies in CHOP-deficient mice, which are less susceptible to amiodarone-induced steatosis [51] and by the study of Kudo et al., in which the administration of 1-(3,4-dihydroxyphenyl)-2-thiocyanate-ethanone (Bix), an enhancer of the ER stress element response, was able to decrease ER stress [52]. With this information, Erez and colleagues demonstrated that Bix administration reduced the amiodarone-induced lipid accumulation by the decrease in sXBP1, CHOP, Cidea and Cidec protein expression in mice [51,53].

Lastly, a wealth of evidence demonstrated that amiodarone induces macrovesicular steatosis upregulating many genes related to lipogenesis, although the specific mechanism of this process remains unknown. In particular, transcripts of SREBP-1, ATP-citrate synthase (ACLY), fatty acid synthase (FAS) and acyl-CoA desaturase 1 (SCD1) were significantly overexpressed after acute or prolonged (24 h or 14 days) amiodarone exposure in HepaRG cells [43].

As regards other antiarrhythmic drugs, such as diltiazem and verapamil, two calcium channel blockers, the data available at present are unclear. Some authors reported that both these drugs are able to induce steatosis in some patients [13,48], but no specific molecular mechanisms have been elucidated yet; therefore, the evidence supporting that diltiazem and verapamil induce steatosis is weak and controversial. In particular, many studies suggested a protective effect of verapamil in different kinds of hepatic damage. For example, Zhou and colleagues showed that intraperitoneal injection of verapamil 25 mg/kg/day for 7 days was able to reduce meta-inflammation, hepatic steatosis and insulin resistance, inhibiting the activation of thioredoxin-interacting protein (TXNIP)/nod-like receptor protein 3 (NLRP3) inflammasome, in mice subjected to a high-fat diet for 10 weeks [54]. Furthermore, in rats fed by high fat diet and 10% ethanol in drinking water, treated with subcutaneous injection of carbon tetrachloride (CCl_4_) to rapidly induce fibrosis, the intragastric administration of 20, 40 and 80 mg/kg/day of verapamil significantly reduced all markers of liver injury and fibrosis such as lipid peroxidation, collagen deposition, α-smooth muscle actin (α-SMA) and transforming growth factor-β1 (TGFβ1) protein expression [55]. Therefore, in light of these data, further studies are needed to better clarify the actual role of verapamil and diltiazem in liver damage.

### 3.2. Antihypertensive Drugs

It is well known that hypertension is often associated with metabolic syndrome with all its manifestation including hepatic steatosis [56]. In particular, recent studies showed a significant interplay between the renin–angiotensin–aldosterone system (RAAS) and NAFLD, demonstrating that hypertension and NAFLD share common pathophysiological pathways. Angiotensin II (Ang II), in fact, promotes insulin resistance, which plays a great part in the onset and progression of NAFLD [57]. Bataller and colleagues showed that human hepatic stellate cells, after activation, synthesize Ang II, promoting the tissue remodeling in the liver [58]. Many studies confirmed that Ang II produced in fibrotic livers stimulates myofibroblast and hepatic stellate cell proliferation, inflammatory cell infiltration and cytokine and growth factor release, such as IL-1β, monocyte chemoattractant protein-1 (MCP-1), TGFβ and connective tissue growth factor [59]. In support of this argument, the infusion of Ang II in a rat model of bile duct ligation increased liver fibrosis and inflammation [60], while RAAS inhibitors administration in animal models of fatty liver attenuated the progression of NAFLD [61,62,63].

Evidence was found in the literature demonstrating the beneficial effects of antihypertensive therapy on NAFLD. For example, losartan, an angiotensin II receptor antagonist used to treat hypertension, showed beneficial effects after 48 weeks of treatment (50 mg/d) in eight hypertensive patients with NASH, leading to a significant decrease in various markers of hepatic fibrosis. In particular, TGFβ1, serum ferritin concentration and serum aminotransferase levels were decreased by losartan administration. The necroinflammation ameliorated in five patients, a reduction in hepatic fibrosis was observed in four patients and iron deposition disappeared in two patients; no side effects were detected in any patient [64].

However, many other antihypertensive agents have been reported to cause liver injuries, even though the precise mechanism involved in the onset of hepatic damage is not clear. Enalapril (ELP) is an angiotensin-converting enzyme (ACE) inhibitor, which is commonly employed for the treatment of cardiovascular diseases, including hypertension and heart failure [65]. It has been reported that mice treated with ELP alone did not develop liver injury. Differently, the combined treatment of ELP with the synthetic glucocorticoid dexamethasone (DEX) and the glutathione synthesis inhibitor L-buthionine-(S,R)-sulfoximine (BSO) resulted in liver steatosis with increased levels of plasma alanine aminotransferase (ALT), accompanied by myeloperoxidase-positive cells infiltrating hepatic tissue and increased oxidative stress-related factors, such as hepatic heme oxygenase-1, serum hydrogen peroxide and hepatic malondialdehyde [65]. Moreover, in a clinical case report, ELP was suspected to induce macro- and microvesicular steatosis in association with neutrophil infiltration and Mallory body formation with satellitosis; the patient had a 10-year history of systemic lupus erythematosus, was under corticosteroid therapy for one year (40 mg/day) and taking ELP (10 mg/day) for 2.5 years to treat hypertension. The hepatic alterations were distributed mostly in zone 1 of the periportal region and less commonly in zone 2 and 3. The extensive periportal necrosis and fibrin deposits, in association with inflammatory cells infiltration, made the clinical features of this patient very close to periportal steatohepatitis, although it was different from both the alcohol-induced and non-alcoholic induced ones, which spread from zone 3 to other zones [66].

As regards nifedipine, a calcium channel blocker, conflicting data emerged from the literature. Babany et al. described, in a clinical case report, the development of steatosis associated with Mallory bodies in a female patient treated with nifedipine and already under treatment with colchicine for gout, with hydroxycarbamide for thrombocythemia and with clonidine for arterial hypertension. After two months of treatment with nifedipine, serum enzymes such as alkaline phosphatase (ALP) and γGT were significantly increased and the liver biopsy showed extensive fibrosis in the portal tracts, micro- and macrovacuolar steatosis affected 50% of the hepatocytes, hepatocyte ballooning and refringent heterogenous clumps were identified as Mallory bodies. After stopping nifedipine administration, all lesions disappeared after one month, without the suspension of other treatment [67]. On the contrary, Nakagami et al. demonstrated that nifedipine, acting as an activator of the PPARγ receptor, reduced NASH in a rat model of methionine and choline deficient (MCD) diet. In fact, rats fed by an MCD diet supplemented by nifedipine for 20 weeks, showed a significant decrease in fibrosis and AST serum levels, when compared with rats fed by an MCD diet alone for the same period [68]. It is known that the activation of PPARγ in the liver is connected with fat deposition in the hepatic parenchyma, whereas the activation of adipose PPARγ protects against steatosis [69]. However, in this regard some clarifications are necessary. Studies by Miyahara et al. demonstrated that PPARγ, in liver, is highly expressed in quiescent hepatic stellate cells (HSC) and that it is downregulated during HSC activation. The administration of PPARγ agonist 15dPGJ2 in cultured activated HSC reduced collagen synthesis, whereas the co-administration of PPARγ agonist 15dPGJ2 with the PPARγ antagonist GW9662 abrogated 70% of the collagen reduction obtained by the agonist alone, showing the potential therapeutic effect of PPARγ ligands in the treatment of liver fibrosis [70]. Similarly, Bae and colleagues demonstrated in HSC-T6 cells that the PPARγ agonist KR62776 administration caused HSC apoptosis, accompanied by a time- and concentration-dependent decrease in the alpha-smooth muscle actin levels, showing that PPARγ stimulation is strictly involved in limiting the activation and proliferation of HSC preventing liver fibrosis [71].

### 3.3. Antibiotics

Among antibiotics, many drugs are able to induce steatosis. For example, tetracycline, a broad-spectrum antibiotic employed to treat various infections of the skin, soft tissues and upper respiratory tract, may induce hepatic steatosis both in humans and in rodents [72]. The family of tetracyclines counts many compounds similar to tetracycline, such as doxycycline, minocycline, methacycline and lymecycline, which induce various types of hepatic injury, including cholestasis, necrosis and microvesicular steatosis [73]. In particular, tetracycline-induced microvesicular steatosis was already observed several decades ago, especially in association with parenteral administration [40,48]. The mechanism involved in the onset of tetracycline-induced steatosis involves the inhibition of fatty acid oxidation genes, such as PPARα, CPT1 and fatty acid binding protein 1 [74,75], and the decrease in hepatic lipoprotein secretion via microsomal triglyceride transfer protein inhibition [76,77]. Moreover, recent studies demonstrated that tetracycline administration affects the expression of many genes associated with fatty acid transport and esterification [43,78]. In particular, Choi et al. demonstrated that 100 μM tetracycline treatment for 24 h upregulates the expression of fatty acid transporter CD36 and diacylglycerol acyltransferase 2 (DGAT2) in HepG2 cells and primary rat hepatocytes, contributing to fatty acid intake and their esterification, leading to the development of steatosis. Similar results were obtained by the same authors in mice treated with 50 mg/Kg/day tetracycline: a significant increase in mRNA and protein levels of CD36 and DGAT2 was observed in livers from tetracycline-treated mice, accompanied by increased levels of hepatic enzymes (AST, ALT, γGT, ALP), serum triglycerides and cholesterol [79]. Furthermore, tetracycline as doxycycline and minocycline could play a role in the progression of steatosis by enhancing oxidative stress generation, via the activating transcription factor 4 (ATF4), which induces ROS generation by means of CYP2E1 upregulation, and the inhibition of the mammalian target of rapamycin (mTOR) [80]. Moreover, Deng et al. demonstrated that tetracycline administration in HepG2 cells produces the alteration of many proteins located in the mitochondria. Among them, the long-chain specific acyl-CoA dehydrogenase, which is one of the key enzymes regulating fatty acid β-oxidation [81]. Lastly, it has been proved that many tetracycline derivatives bind to the mitochondrial ribosomes so inhibiting mtDNA translation [73].

**Figure 3:**
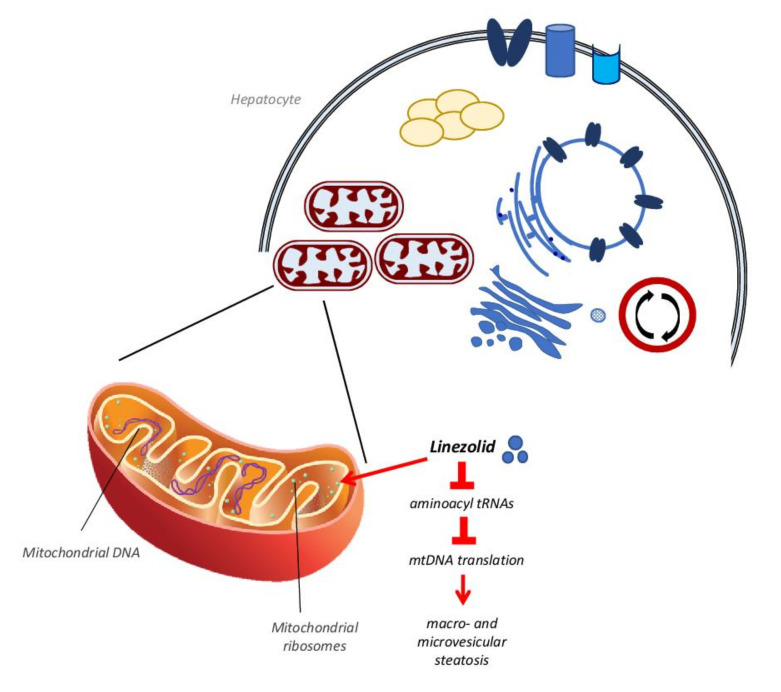
Linezolid activity on mitochondrial ribosomes. Linezolid stops the protein synthesis in bacteria by binding their ribosomes. At the same time, this drug can bind human mitochondrial ribosomes inhibiting the bond of aminoacyl tRNAs, blocking the mtDNA translation and reducing the mitochondrial respiration chain complexes activity. This process, after several weeks, produces micro and microvacuolar steatosis.

This last mechanism of action is also typical of another antibiotic, the oxazolidinone linezolid, which is used to treat multi drug-resistant pathogens such as Gram-positive Enterococcus faecium, Staphylococcus aureus and Mycobacterium tuberculosis [82]. Linezolid binds to a site on the bacterial 23S ribosomal RNA of the 50S subunit, which prevents the formation of a functional 70S initiation complex, so affecting protein synthesis [82]. At the same time, this mechanism can also target mammalian mitochondrial protein synthesis via its binding to mitochondrial ribosomes and causing clinical manifestations observed in patients with genetic defects of OXPHOS [40,83]. This occurs because mitochondrial mammalian ribosomes share some similarities with bacterial ribosomes at the level of the linezolid binding site [82,84], so when the drug interacts with mammalian ribosomes it may affect mitochondrial protein synthesis, resulting in the reduction of the mitochondrial respiration chain activity [82,85,86,87] (Figure 3). This condition, after several weeks of treatment with linezolid, produces in many patients an increase in plasma transaminase levels as well as macro- and microvesicular steatosis and bile duct injuries [48,83,85,88].

As regards rifampicin, an antibiotic drug largely used in the therapy of tuberculosis, it has been demonstrated that it can induce hepatic lipid accumulation. Huang et al. showed that after 200 mg/Kg/day administration of rifampicin, mice displayed increased serum levels of ALT and hepatic triglycerides already after 3 days of treatment; these parameters increased in a more significant way after 4 weeks of drug administration [89]. The authors demonstrated that rifampicin induces lipid hepatic accumulation through the upregulation of both fatty acid synthesis and hepatic lipid uptake. In particular, rifampicin administration was found to upregulate mRNA expression of SCD1, ACC and FAS, concomitantly with the upregulation of PXR and PPARγ. In addition, it was also demonstrated that rifampicin upregulates CD36, hepatic FATP, a fatty acid transporter that participates in the uptake of long-chain fatty acids [90] and LDLR, a surface receptor that removes cholesterol-carrying LDL from plasma by receptor-mediated endocytosis [91]. Similar data were obtained by Allard et al., which demonstrated that rifampicin induces a dose-dependent lipid accumulation in HepaRG cells, through mtFAO inhibition [92].

### 3.4. Antineoplastic Drugs

Many antineoplastic drugs are able to induce DILI and DISH. Among them, tamoxifen is well known to induce hepatic steatosis and steatohepatitis. Tamoxifen is classified as an antagonist of the estrogen receptor and acts mainly in breast tissue. It is used as an adjuvant treatment for breast cancer and it decreases cancer recurrence and mortality in women with estrogen receptor-positive tumors [93,94]. It has been proven that about 40% of patients receiving long-term tamoxifen administration develop steatohepatitis [95]. The estimated time to the onset of steatosis following tamoxifen administration is approximately 22 months, but the steatosis incidence before 2 years of treatment is similar to the placebo, as demonstrated in a prospective, randomized, double blind, placebo-controlled trial, recruiting 5408 women subjected to hysterectomies from 58 centers in Italy. The 20 mg/day tamoxifen administration was correlated with an increased risk of NASH development only in individuals who were overweight, or who showed obesity and metabolic syndrome [96]. In according with these results, many other studies reported that tamoxifen is a risk factor to develop steatosis when the patient is already affected by metabolic syndrome or diabetes [95,96,97,98]. As regards the mechanism of tamoxifen-induced hepatic steatosis, a lot of evidence, obtained both in animal models [99,100] and in postmenopausal women treated with estrogens [101,102,103], demonstrated that estrogens play beneficial effects on liver metabolism, enhancing insulin sensitivity and preventing lipid accumulation and steatohepatitis. Thus, tamoxifen being an estrogen receptor antagonist, it promotes DISH [53]. In particular, it has been studied that this effect is strictly linked to the activity of estrogen receptor ERα; in fact, ERα knockout mice showed lipid intrahepatic accumulation due to decreased insulin sensitivity [104]. On the contrary, the administration of ERα receptor agonists decreased steatosis in aromatase knockout mice [105]. The estrogen receptor ERβ also seems to be involved in tamoxifen-induced steatosis; in fact, recent evidence demonstrated that the administration of the ERβ selective agonist isoquinolinone LGND2 prevented steatosis in mice fed by high-fat diet or by a methionine and choline deficient diet [106]. Moreover, a wealth of evidence demonstrated that estrogen receptors ERα and ERβ are also expressed on the mitochondrial surface [107]. Their activation can affect beta oxidation [108] upregulating specific genes such as PPARα and PPARγ, beta-3-hydroxyacyl CoA dehydrogenase (HADHβ), CPT1 and pyruvate dehydrogenase kinase 4 (PDK4), as demonstrated in rats treated with estrogen [109]. In particular, Zhou et al. demonstrated that ERα direct interaction with HADHβ increases cellular beta oxidation and that tamoxifen inhibits this association leading, to a reduced beta oxidation rate [110]. Apart from its activity on estrogen receptors, other studies in mice revealed that tamoxifen accumulates in the mitochondrial matrix, impairing fatty acid oxidation, respiration, and mitochondrial DNA relaxation and synthesis due to its chemical properties as a DNA-intercalating and cationic amphiphilic compound [111]. Furthermore, Lelliot et al. demonstrated a tamoxifen-induced microvesicular steatosis and marked hypercholesterolemia in rats after only 5 days of treatment. In addition, tamoxifen downregulated fatty acid synthase (FAS) expression, as demonstrated by the accumulation of malonyl-CoA, causing the stop of fatty acid oxidation leading to steatosis [112]. On the contrary, more recent in vitro studies on HepG2 cells demonstrated that tamoxifen induction of macrovesicular steatosis is mediated by an enhancement of fatty acid synthesis through the induction of SREBP-1c and its downstream effectors, such as FAS, ACC and SCD [113]. Lastly, studies on breast cancer patients undergoing tamoxifen treatment demonstrated that there is an association between a particular polymorphism in the gene of cytochrome P450C17alpha, related to the circulating levels of estrogens, and the development of tamoxifen-induced hepatic steatosis [114].

Toremifene is another selective estrogen receptor modulator, also used as an adjuvant treatment for breast cancer. Toremifene is structurally similar to tamoxifen, apart from the substitution of a hydrogen atom with chlorine on the ethyl side chain; this change seems to be related to fewer thromboembolic events and reduced association with endometrial cancer [115]. As regards toremifene-induced steatosis and steatohepatitis, only a few cases of liver damage have been described after its administration. In particular, Hamada et al. demonstrated that only 7.7% of patients developed fatty liver after 3–5 years of toremifene administration. Moreover, toremifene-induced steatosis was less severe than that observed after tamoxifen administration; in addition, toremifene did not alter AST, ALT, γGT and total cholesterol serum levels [116]. The incidence of toremifene-induced NASH was also lower in comparison with tamoxifen [116].

Irinotecan is an antineoplastic compound often used in combination with other agents, as oxaliplatin, 5-fluorouracil (5-FU) and leucovorin, in the treatment of digestive cancers such as colorectal and pancreatic tumors. Its cytotoxic effect is due to its activity as a topoisomerase I complex inhibitor, which causes single-stranded DNA lesions and apoptotic cell death [94]. Unfortunately, one of the side effects of irinotecan is related to the development of steatohepatitis in about 20% of colorectal tumor patients treated with this compound before undergoing hepatic metastasis resection. This event results in a significant increase in 90-day post-operative mortality [117,118]. The mechanism whereby irinotecan induces steatohepatitis remains unknown, but it has been hypothesized that a mitochondrial dysfunction may be involved. In fact, irinotecan, like tamoxifen, intercalates into the mtDNA leading to the downregulation of enzymes involved in electron transport; in addition, as a cationic amphiphilic drug, it also uncouples the mitochondrial oxidative phosphorylation [18]. This situation is followed by an increase in ROS production, lipid peroxidation and beta-oxidation, which lead to the Kupffer cell-mediated release of pro-apoptotic cytokines, such as TNF-α, and pro-fibrotic factors, such as TGFβ, resulting in cell death, inflammation and fibrosis [119].

Additionally, 5-fluorouracil (5-FU) has been listed in the category of drugs able to induce steatosis. 5-FU is one of the most used anti-cancer compounds to treat digestive, head and neck, ovary and breast tumors. It is an anti-metabolite, in particular an anti-pyrimidine, which blocks the DNA and protein synthesis [94]. Similar to other antineoplastic compounds, it can cause lipid accumulation, especially in metastatic colorectal cancer patients, in which steatosis incidence range is registered between 30% and 47% [120]. Moreover, it has been demonstrated that 5-FU is related to chemotherapy-associated steatohepatitis (CASH), which is often observed in patients treated with combined therapy of 5-FU and irinotecan [121,122,123]. Recently, the possible mechanism of the 5-FU-mediated induction of DISH has been elucidated by Sommer et al. These authors, using HepG2 cells and primary human hepatocytes, demonstrated that 5-FU induces a significant increase in hepatic triglyceride accumulation, accompanied by dysregulation of mitochondrial function and an increase in fatty acid acyl-CoA oxidase 1 (ACOX1), which represents the first step of the peroxisomal beta-oxidation. ACOX1 generates ROS, inducing the upregulation of the antioxidant enzyme heme oxygenase 1 (HMOX1) in 5-FU treated cells, followed by a significant increase in the activation of c-Jun N-terminal kinase (JNK) and of pro-inflammatory genes IL-8 and ICAM-1. The same authors obtained comparable data also in an in vivo model, showing that ACOX1 and HMOX1 upregulation, as well as JNK- and pro-inflammatory gene expression and immune cell infiltration, induce NASH in mice [124]. Regarding the JNK trigger, recent studies demonstrated that an interplay between activated JNK (P-JNK) and mitochondria occurs through the SH3BP5 (SAB) protein. SAB is expressed in the mitochondrial outer membrane where it acts as a P-JNK docking protein and as a substrate of JNK [125]. JNK-induced SAB phosphorylation promotes mitochondrial dysfunction and ROS production and, as a feedback reaction, it activates the upstream mitogen-activated protein kinase (MAPK) pathway, which is the initial event in the P-JNK-mitoSAB-ROS activation loop [126,127,128]. In addition, studies in mice fed by high-fat, high calorie and high-fructose (HFHC) diet demonstrated a time-dependent increase in SAB expression, with the concomitant JNK activation [129]. The same authors also demonstrated that inducible hepatocyte specific SAB deletion (Sabi∆Hep) in mice reduced significantly the JNK activation after 8 weeks of HFHC diet, accompanied by decreased body fat after 16 weeks. Similar data were obtained in 40-week-HFHC fed mice treated with SAB N-acetylgalactosamine antisense oligonucleotide (GalNAc-Sab-ASO) to obtain SAB knockdown mice. SAB knockdown mice, after 40 to 52 weeks of HFHC diet, showed a decreased steatohepatitis and fibrosis and restored SAB and P-JNK levels, with respect to those found in mice fed by standard diet, demonstrating the crucial role of P-JNK-mitoSAB-ROS loop in NASH [129].

Finally, it has been proposed that the onset of steatosis in patients treated with 5-FU could be attributed to the individual expression pattern of specific genes linked with 5-FU metabolism, in particular to decreased levels of dihydropyrimidine dehydrogenase (DPD) [130]. Presumably, some catabolites obtained by DPD activity, such as fluoro-beta-alanine, remain after the interruption of 5-FU therapy in the hepatocytes and, saturating the metabolic pathway, causing the hepatic lipid accumulation [131].

The antineoplastic drug L-asparaginase is a protein-based enzyme obtained from Escherichia coli which hydrolyses asparagine, an amino acid not synthesized endogenously by leukemic cells, to aspartic acid and ammonia causing a decrease in asparagine serum levels [132] and therefore depriving cancer cells of this essential compound. It is employed to treat acute lymphoblastic leukemia and, like other anti-cancer agents, it is associated with the onset of hepatic steatosis in 40–87% of patients within 9 months after the first administration [94]. As regards the mechanism involved in the onset of L-asparaginase-induced steatosis, there is not much information available to date. There is some evidence showing that L-asparaginase may reduce protein synthesis and the level of both asparagine and glutamine, leading to hepatic micro- and macrovesicular steatosis [133,134,135,136]. In particular, Bodmer et al. in a clinical case report described a patient suffering from acute lymphoblastic leukemia and treated with L-asparaginase, which developed hepatic microvesicular steatosis accompanied by liver mitochondrial dysfunction and alterations in very-low-density lipoprotein (VLDL) metabolism and secretion [137]. The authors suggested that mitochondrial dysfunction could be attributed to the decreased hepatic protein synthesis, which is responsible for the lack of essential mitochondrial beta-oxidation proteins; in addition, the L-asparaginase-induced reduction in serum glutamine levels also was hypothesized to have a role [134,135,138]. In fact, glutamine is important for glutathione (GSH) synthesis and depletion of GSH stores is one of the leading causes of mitochondrial injury induced by oxidative stress in hepatocytes [139,140]. Moreover, in this case report a decrease in triglyceride serum levels, associated with low levels of VLDL, was found after L-asparaginase administration [137]. Probably, this change results from the asparaginase-induced inhibition of hepatic protein synthesis, which also may decrease the synthesis of microsomal triglyceride transfer protein (MTP), one of the key proteins in the VLDL formation and secretion mechanism, as demonstrated by Raabe et al.. In this paper, MTP knockout mice developed microvesicular steatosis caused by the absence of VLDL formation and secretion [141].

Methotrexate (MTX) is a folic acid antagonist employed as an antiproliferative and immunosuppressant agent in the treatment of malignancies and autoimmune diseases. It is employed in head, neck, bladder, lung, gynecologic cancers, lymphomas and leukemias; moreover, its use is recommended for the treatment of psoriatic and rheumatoid arthritis and in Crohn’s disease [94]. It has been demonstrated that MTX induces lipid hepatic accumulation with a pathological pattern typical of alcohol-induced steatosis, mostly in patients chronically treated with low doses of MTX [53]. Moreover, in about 20% of patients, simple steatosis evolves into cirrhosis; to prevent this complication, guidelines recommend to perform a biopsy before and during the therapy with MTX [142], because it is one of the major causes of DISH. The mechanism by which MTX induces hepatic steatosis or cirrhosis is not completely clear; however, some evidence-based theories have been proposed. MTX affects oxygen uptake in the mitochondria, causing the decrease in oxidative phosphorylation [143]. MTX also downregulates many mitochondrial enzymes, such as 2-oxoglutarate, isocitrate, malate, and pyruvate dehydrogenases [144]. In addition, available data show that MTX induces the depletion of mitochondrial folate stores; in fact, it enters mitochondria and prevents the replenishment of folic acid [145]. Some evidence demonstrated that MTX, through ROS generation, impairs the mitochondrial membrane potential and induces apoptotic cell death by caspase activation [146], leading to mitochondrial dysfunction [147]. Moreover, Song et al. demonstrated that MTX induces steatohepatitis through its toxicity on the gastro-intestinal tract. Through the disruption of the intestinal barrier function, MTX favors bacterial translocation from the gut to the liver, as shown by rats treated with 3.5 mg/kg MTX for three days and then administered orally with E. coli expressing green fluorescent protein [148]. Lastly, it has been reported that MTX also induces fibrosis through the activation of the adenosine pathway. In particular, fibroblasts treated with 100 nM MTX release a three-fold increased amount of adenosine [149], which over-activates the adenosine A2A receptor on the hepatic stellate cells, resulting in increased levels of collagen and downregulation of matrix metalloprotease activity [150].

### 3.5. Antiepileptic Drugs

Valproic acid (VPA) is an eight-carbon fatty acid molecule, administered as oral or parenteral formulations. VPA has been commonly applied in the treatment of epilepsy [151], both in adults and in children, since 1967. It has been estimated that more than one million people worldwide take VPA every day [152]. In fact, besides being the first line compound for newly identified epileptic disorders, it is also used to treat neuropathic pain, migraine [153], and bipolar disorder. Moreover, it is reported to be effective for spinal muscular atrophy [154], leukemia [155] and some solid tumors [156,157]. Nevertheless, VPA treatment can cause several adverse effects, such as dizziness, tremor, nausea, endocrinological disorders, obesity, insulin resistance, weight gain, and hepatotoxicity [158]. VPA is mainly metabolized at the hepatic level through three pathways: (i) glucuronidation, (ii) mitochondrial β-oxidation, (iii) cytochrome P450-mediated ω-oxidation. These metabolic pathways produce about 50 known metabolites, some of which are biologically active and hepatotoxic [159]. VPA-induced hepatotoxicity is not easy to be diagnosed because patients are often asymptomatic. Even though VPA-hepatotoxicity has three clinical forms, which are hyperammonemia, acute hepatocellular injury and Reye-like syndrome, all of these forms present mitochondrial injury and microvesicular steatosis [160]. Several mechanisms have been suggested for VPA-induced hepatic steatosis. Both VPA and its metabolite 4-ene-VPA can sequester Acyl CoA, making it unavailable for β-oxidation. The complex VPA-CoA, by inhibiting the binding of both malonyl-CoA and palmitoyl-CoA to CPT1, attenuates fatty acid transport into mitochondria [161]. VPA also inactivates α-ketoglutarate dehydrogenase, reducing citric acid cycle flux. Furthermore, high concentrations of VPA metabolites have an inhibitory effect on pyruvate uptake into rat liver mitochondria, preventing mitochondrial oxidative phosphorylation [162]. As a consequence, a 48-h VPA treatment has been associated with a reduction in the oxygen consumption rate, mitochondrial membrane potential and ATP levels in HepG2 cells. A prolonged VPA exposure not only decreases basal respiration rates in a collagen sandwich system of primary human hepatocytes [163], but it also favors lipid accumulation, due to the increased expression of the fatty acid transporter CD36 through epigenetic modification [164]. Besides mitochondrial damage, VPA administration correlates also with increased levels of oxidative stress and decreased antioxidant defense. On one hand, lipid hydroperoxide (LPO) and TBAR concentration increase in plasma and liver tissue in a VPA concentration-dependent way; on the other hand, VPA causes rapid hepatocyte GSH [165] and Nrf2 depletion [166]. Increased VPA-hepatotoxicity has been associated with genetic polymorphism of specific genes, including polymerase γ gene (plog), glutathione S-transferase (gst), superoxide dismutase 2 (sod2), and metabolizing enzymes. Different approaches focused on the reduction of VPA hepatotoxicity are under investigation. For instance, it has been demonstrated in animal and clinical studies that L-carnitine supplementation may attenuate this adverse effect. Another strategy employed to neutralize VPA hepatotoxicity consists in the administration of pantothenic acid, a precursor of CoA. A protection against oxidative stress could be provided with antioxidants, such as N-acetylcysteine, a precursor of GSH, or zinc and selenium [167]. Recently, in VPA-induced NAFLD model in mice, both oxidative stress and steatogenic effect were reversed by the administration of obeticholic acid (OCA), a farnesoid X receptor (FXR) activator [168].

As regards other antiepileptic drugs, evidence does exist about their possible involvement in the onset of steatosis or steatohepatitis. For example, in a case report it has been shown that an epileptic woman, treated with carbamazepine for three months, developed both porphyria and steatohepatitis. The authors suggest that, in this case, the hepatic dysregulation of fatty acid content accompanied by inflammation may be due to the formation of a carbamazepine toxic metabolite which impaired the mitochondrial membrane of hepatocytes, decreasing the microsomal cytochrome P-450 dependent enzyme activity [169]. This hypothesis came from some experimental evidence, in which it was demonstrated that carbamazepine metabolites can make a covalent bond to the hepatic microsomes, inducing a toxic reaction [170]. However, the data obtained from a cross-sectional study including 130 patients suffering from idiopathic epilepsy reveals that only the VPA seems to alter significantly the serum lipid levels, whereas other drugs such as carbamazepine and lamotrigine show no effects [171].

### 3.6. Glucocorticoids

Synthetic glucocorticoids (GCs), such as dexamethasone, betamethasone, prednisolone and triamcinolone, control various physiological functions including development, homeostasis, metabolism, cognition and inflammation [172] and are generally employed for their anti-inflammatory properties at high doses and prolonged periods. Glucocorticoids are among the most widely prescribed medicaments, so much so that the global income that revolves around the sale of glucocorticoids amounts to more than 10 billion dollars/year [173]. In particular, glucocorticoids are used in the treatment of many clinical manifestations such as asthma, allergy, septic shock, rheumatoid arthritis, inflammatory bowel disease, and multiple sclerosis [174]. Unfortunately, the prolonged use of GCs can produce many side effects, including insulin resistance, hepatic lipid deposition and steatosis [175]. It is well known that one of the most immediate effects of GC administration is an increased appetite, which may result in weight gain. This initial condition is followed by hepatic fatty acid deposition, dyslipidemia, insulin resistance, muscle wasting, hypertension, skin and skeletal fragility and psychological disorders. All these abnormalities are commonly observed in patients suffering from Cushing’s syndrome, who display elevated cortisol levels [176,177]. Studies have been conducted to shed light on the mechanisms involved in the onset of these symptoms. For example, it has been demonstrated that GC levels above the physiological threshold induce hyperphagia and obesity, probably due to a reduced sensitivity to leptin [178,179,180]. In fact, it has been reported both in Huh7 cells and in rats, that the leptin bound to its receptor OBRb initiates a tyrosine cascade phosphorylation after JAK/STAT signaling activation [181]; this pathway is blocked by GCs [179]. Moreover, after GC prolonged administration, insulin resistance and elevated glucose occur, leading to ChREBP and SREBP-1c increase. These events promote the GC-mediated increase in the de novo lipogenesis, upregulating genes such as ACC and FAS [182]. SREPB-1c is also involved in the inhibition of PPARγ coactivator 1α (PGC-1α), which activates phosphoenolpyruvate carboxykinase (PEPCK), a rate-limiting enzyme that promotes gluconeogenesis [183]. At the same time, the increased glucose disposal favors the synergistic effect of ChREBP and SREBP-1c [184]. Lastly, recent studies reported that, after GC administration, an up-regulation of mitogen-activated protein kinase phosphatase-3 (MKP3) plays a critical role in GC-induced hepatic de novo lipid deposition. In fact, mouse hepatoma cells (Hepa1–6) treated with 2.5 μM dexamethasone for 2 h, displayed increased expression of MKP3 via the forkhead box protein O1 (FOXO1) and the same result was obtained in lean mice treated with 15 mg/kg dexamethasone for 28 days. The role of MKP3 was also confirmed using MKP3 knockout (MKP−/−) mice versus wild type (WT) mice. Dexamethasone injection caused a significant increase in the bodyweight of WT mice after five weeks of injection, when compared with vehicle-treated mice. On the contrary, no weight gain was observed in MKP3−/− mice. In addition, dexamethasone-induced triglyceride hepatic accumulation was totally abolished in MKP3−/− mice, when compared with WT mice. It was also shown that, in MKP3−/− and not in WT mice, dexamethasone injection did not increase the expression of lipid synthesis genes, such as PPARγ, FAS, SCD1 and ACC [185].

Another mechanism by which GCs cause NAFLD is through the increased uptake of free fatty acid in the liver by the upregulation of CD36 fatty acid transporter [175]. In fact, studies by D’souza and colleagues demonstrated that the subcutaneous implantation of four 100 mg-corticosterone pellets or placebo in rats for 16 days induced a CD36 expression increase when the pharmacological treatment was associated with both a high-fat diet and a standard diet [186].

Moreover, GCs administration causes hepatic lipid accumulation by causing a decreased secretion of lipids in form of very low density lipoproteins (VLDL). In particular, both in vitro and in vivo experiments demonstrated that dexamethasone administration affects the stability of mRNA of hepatic triacylglycerol hydrolase (TGH), the lipase that hydrolyses triacylglycerol (TAG) before its re-esterification to form VLDL. On the other hand, dexamethasone upregulates the expression and activity of diacylglycerol acyltransferases 1 and 2, favoring the de novo TAG synthesis [187]. Thus, by increasing the de novo lipogenesis and decreasing the utilization of stored TAG for VLDL synthesis, GC administration produces further hepatic lipid droplet formation and steatosis onset and progression.

**Figure 4:**
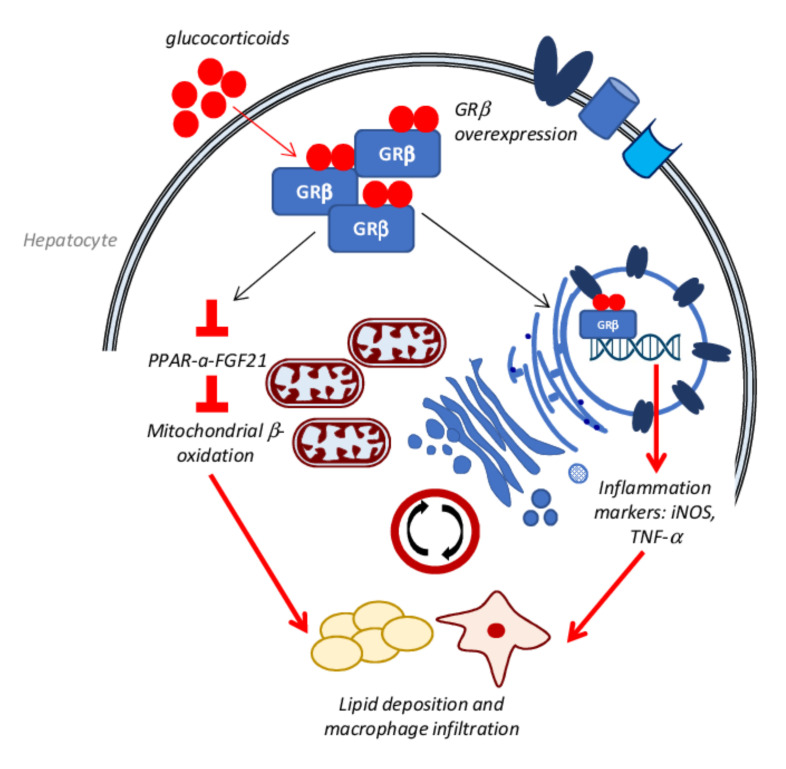
Schematic representation of glucocorticoid receptor β (GRβ) contribution in hepatic lipid deposition. GRβ overexpression induces both the inhibition of the PPARα-FGF21 signal pathway, decreasing β-oxidation of fatty acid, and at the same time is involved in inflammatory process establishment by the secretion of TNF-α and upregulation of iNOS protein expression. This condition leads to further lipid deposition and macrophage hepatic infiltration.

An additional mechanism contributing to GC-induced hepatic steatosis is the decreased beta-oxidation rate, caused by a decreased activity of the PPARα-FGF21 axis, as seen in high-fat-fed mice overexpressing glucocorticoid receptor β (GRβ) at the hepatic level [175]. Moreover, at the same time, GRβ overexpression is involved in inflammatory process activation by the secretion of TNF-α and inducible nitric oxide synthase (iNOS) upregulation. These events lead to macrophage infiltration in the liver and the further increase in lipid deposition and NAFLD progression [188] (Figure 4).

The transcriptional coactivator of the glucocorticoid receptor MED1/PPARBP [189] is also necessary to induce steatosis. In fact, a conditional gene disruption of MED1 in mouse liver resulted in the loss of dexamethasone ability to induce hepatic steatosis [189].

Lastly, it has been recently demonstrated that angiopoietin-like 4 (ANGPTL4, fasting-induced adipose factor), a primary downstream gene of GC receptor signaling pathway in both hepatocytes and adipocytes, is strictly linked with the GC-induced hepatic steatosis and hypertriglyceridemia. In Angptl4 knockout mice (Angptl4−/−), despite the administration of dexamethasone, the rate of hepatic steatosis and hypertriglyceridemia were significantly decreased [190].

### 3.7. Nonsteroidal Anti-Inflammatory Drugs (NSAIDs)

Nonsteroidal anti-inflammatory drugs (NSAIDs) are among the most prescribed medications in the world for their analgesic, anti-inflammatory and antipyretic properties [191]. It is estimated that more than 30 million people consume NSAIDs every day, and this number will probably continue to increase over time. Despite the efficacy and the spread of NSAIDs, they cause a plethora of side effects [192]. In about 60–70% of patients, long-term administration of NSAIDs is related to the development of mucosal damage [193,194,195], as alteration of intestinal permeability, inflammation, erosion of gastrointestinal epithelia, protein loss, anemia, bleeding [196,197]. Many NSAIDs are listed in the category of drugs inducing steatosis. Contradictory data were published on aspirin or salicylic acid. In some studies, aspirin was found to cause liver damage through the induction of mitochondrial dysfunction, which leads to metabolic abnormalities and microsteatosis as part of the clinical picture of Reye’s syndrome. Reye’s syndrome is an acute encephalopathy characterized by liver damage, which is one of the aspirin side effects in children. For this reason aspirin is replaced by ibuprofen or paracetamol in pediatric patients [198]. Moreover, experimental studies conducted in isolated rat hepatocytes suggest that salicylic acid administration caused hepatic cell death mediated by mitochondrial dysfunction after dose-dependent ATP depletion and lipid peroxidation [199]. On the other hand, in rats fed with a choline-deficient, L-amino acid-defined (CDAA) diet to induce NASH, 150 mg/kg/day of aspirin significantly reduces liver steatosis, inflammation and fibrosis [200]. Similarly, Ibrahim and colleagues demonstrated that the administration of NO-aspirin in cholesterol-diet fed rats significantly decreases hepatic triglycerides, malondialdehyde and nitric oxide, by reducing inducible nitric oxide synthase (iNOS) and cyclooxygenase-2 (COX-2) [201]. Different possible molecular mechanisms have been proposed to explain the potential protective role of salicylic acid in NAFLD. It is well known that aspirin acts on various targets, which can all play a beneficial role in NAFLD [202]. Firstly, aspirin induces the expression of endothelial nitric oxide synthase (eNOS) and vascular endothelial growth factor (VEGF), resulting in an antioxidant response [203]; then, the aspirin-mediated inhibition of iNOS and TNF-α decreases inflammation and fibrosis [204]. Aspirin also reduces the expression of platelet-derived growth factor (PDGF)-C, through the inhibition of TGFβ-regulate profibrotic pathway [205]. Lastly, aspirin inhibits the activation of the mitogen-activated protein kinase (MAPK) pathway induced by PDGF, so promoting a reduction in Jun N-terminal kinase (JNK) activity, which leads to the initiation of the insulin signaling pathway, ameliorating insulin resistance [206]. Data about aspirin-induced protection from NAFLD were also obtained in patients; in fact, it has been demonstrated in a cross-sectional study that the use of aspirin is associated with a lower prevalence of NAFLD in general US population, in particular in men and elderly patients [202]. More recently, Simon et al. demonstrated in a prospective cohort study of 361 adults suffering from NAFLD, that daily use of aspirin reduces the histologic features of NAFLD and NASH and the risk of progression to advanced fibrosis [207]. Thus, further investigations are needed to completely clarify the role of aspirin in NAFLD development.

Acetaminophen (APAP) is the most used analgesic and antipyretic agent in the world since its first introduction in clinical practice in 1955. It is considered a safe drug, but it may cause fatal acute liver failure when the dose exceeds 4 g/day. Several studies reported that its administration is more dangerous in patients already suffering from NAFLD, NASH or obesity. APAP-induced liver damage is related to the CYP3A4 and CYP2E1-mediated generation of its metabolite N-acetyl-p-benzoquinone imine (NAPQI), which is detoxified by glutathione (GSH) [208]. In already compromised hepatocytes, as they are in NAFLD or NASH, GSH stores are poor because of the increased activity of CYP450, in particular CYP2E1, which is post-transductionally upregulated by insulin. In fact, in primary hepatocytes isolated from high-fat diet-fed rats, the administration of APAP causes an excess of NAPQI production and the subsequent generation of ROS and adducts in proteins and other biomolecules, leading to mitochondrial damage and further depletion of GSH stores [209]. Moreover, in mice, after APAP intoxication, an accumulation of acylcarnitine in serum and liver occurs [210,211] correlated with a concomitant intrahepatic lipid deposition and an inhibition of mitochondrial long-chain fatty acid oxidation [212]. This mechanism could explain the APAP-induced microvesicular steatosis onset in rodents. It has been suggested, in fact, that NAPQI probably binds in a covalent way to one of the enzymes involved in long-chain fatty acid oxidation [48] or, alternatively, it can interact with MRC, leading to an accumulation of long-chain acylcarnitines [213]. Lastly, studies by Li et al. demonstrated that APAP intoxication significantly reduces hepatic PPARα expression via the stabilization of hypoxia inducible factor-1α (HIF-1α), leading to mtFAO impairment [214,215].

**Figure 5:**
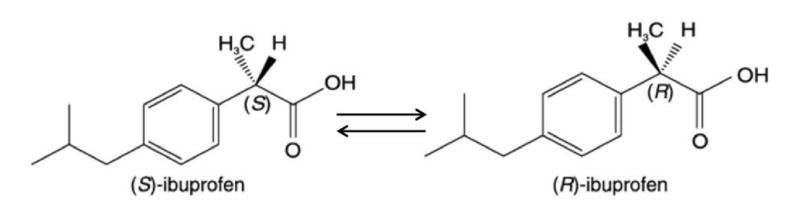
Ibuprofen S^+^ and R^-^ structure. The majority of NSAIDs are commercialized as the racemate mixture.

A similar mechanism of action was attributed also to pirprofen and ibuprofen [216,217]. Both drugs are 2-arylpropionates with an asymmetric carbon that produces two enantiomers, the S^+^ and R^-^ forms. Except for naproxen, this category of anti-inflammatory drugs is commercialized as racemate mixtures [217] (Figure 5).

It was believed for a long time that only the S^+^ enantiomer of ibuprofen had anti-inflammatory properties [218], while the R^-^ enantiomer formed an acyl coenzyme A derivative that was incorporated into lipids [219,220] and inhibited mitochondrial oxidation of natural coenzyme A. However, it has been proven that both enantiomers can inhibit mtFAO in vitro and in vivo, and that the R^-^ enantiomer can stereoselectively bind to coenzyme A in vitro, when CoA is at low concentration, so inhibiting long chain fatty acids mitochondrial uptake and beta oxidation [217]. It has been demonstrated that pirprofen also induces microvesicular steatosis through mtFAO inhibition. In fact, 2 mM pirprofen administration to rat mitochondria decreased by 50% the formation of [^14^C]acid-soluble beta-oxidation products and reduced by 70% the formation of [^14^C]CO_2_ when hepatic mitochondria were incubated with [^14^C]palmitic acid, ATP, carnitine and coenzyme A. The same authors obtained similar data after 2 mmol/kg pirprofen administration in vivo, observing also a doubled deposition of triglycerides in the liver tissue [216]. Lastly, a recent study from Lu and colleagues revealed that ibuprofen inhibits the transcriptional activity of farnesoid X receptor (FXR) in HepG2 cells [221]. It is known that FXR is one of the most important regulators of hepatic triglycerides, so its inhibition by ibuprofen could be related to the onset of steatosis after ibuprofen administration.

Diclofenac, a phenylacetic acid, and naproxen, a propionic acid, have been reported to induce microvacuolar steatosis through the inhibition of mtFAO. In particular, diclofenac inhibits the glutamate/malate-driven respiration on mitochondria isolated from ob/ob mice [222], while naproxen has been proposed to inhibit β-oxidation of short- and medium-chain fatty acids [223].

Few data have been published in the literature about ketoprofen-induced NAFLD; in a case report by Famularo and colleagues, the administration of this drug was associated with fatty acid deposition in the hepatic parenchyma, accompanied by cholestasis and transaminase release. This clinical picture suggests that ketoprofen may induce the inhibition of mitochondrial function with consequent accumulation of lipids and ROS, lipid peroxidation, hepatocyte apoptosis and necrosis and dysregulation of bile secretory machinery [224].

### 3.8. Nucleoside Reverse Transcriptase Inhibitors (NRTIs)

Nucleoside reverse transcriptase inhibitors (NRTIs) include many drugs, such as zidovudine, didanosine, stavudine, lamivudine, zalcitabine, abacavir and tenofovir used for the treatment of virus infections from the human immunodeficiency virus (HIV) or the Hepatitis B virus (HBV). Some NRTIs can induce many side effects including hepatic steatosis [225]. In particular, two cross-sectional studies conducted in patients affected by HIV demonstrated that the prevalence of NAFLD, in patients treated with NRTIs, ranges from 31% to 37% [226,227]. NRTIs are 2′,3′-dideoxynucleosides without the hydroxyl group in 3′ position of the sugar ring; once incorporated into the viral DNA chain, they inhibit its elongation, blocking the reverse transcriptase enzymes [228]. However, they can act also as substrates for human DNA polymerases and, in particular, they can inhibit the human DNA polymerase γ, the only one able to replicate the mitochondrial DNA [229,230], leading to mitochondrial dysfunction and subsequent hepatotoxicity, hepatic steatosis and lactic acidosis. NRTIs mostly involved in this kind of mechanism are zidovudine, stavudine and didanosine [231].

Zidovudine (3-azido-3′-deoxythymidine or AZT), was the first drug approved by US Food and Drug Administration for the treatment of AIDS and it is still the most employed compound to treat HIV infected patients [232]. However, many patients infected by HIV and treated with AZT develop lethal liver abnormalities, such as lipid dysregulation and accumulation, steatohepatitis and acidosis [14,15,16,17]. Studies conducted in mice demonstrated that the mechanism of AZT-induced fatty acid accumulation in the hepatic parenchyma is due to AZT-induced oxidative and endoplasmic reticulum stress. In fact, C57BL/6J female mice administered intraperitoneally with 400 mg/Kg/day AZT for 10 days, displayed a significant increase in hepatic triglyceride levels and inflammation markers, such as lipid peroxidation and protein modification, as well as in ER stress markers, such as the molecular chaperone glucose-regulated protein 78 (GRP78) and its downstream targets, including protein kinase-like endoplasmic reticulum kinase (PERK) and the eukaryotic translation initiation factor-2α (eIF2α). Moreover, these mice displayed increased levels of nuclear SREPB-1c, while PPARα, phospho-AMP kinase and 3-keto-acyl-CoA thiolase were significantly decreased, suggesting that these alterations in fatty acid metabolism could be part of the mechanism by which AZT induces steatosis [232].

Another mechanism by which NRTIs can induce steatosis is the inhibition of autophagy. Stankov and colleagues demonstrated, using HepG2, Huh7 and primary human hepatocytes, that both AZT and stavudine inhibit constitutive and induced autophagy in a dose- and time-dependent manner. This occurrence causes mitochondrial dysfunction, ROS accumulation and augmented apoptosis, accompanied by increased lipid deposition within the cells, contributing to NAFLD in HIV-infected patients [233]. Finally, it has been reported that, in a clinical picture of HIV/HCV coinfection, the combined therapy with didanosine and ribavirin caused mitochondrial toxicity and consequent lipid deposition, due to reduced didanosine phosphorylation and its cytosolic accumulation [234]. Didanosine itself also leads to a dose-dependent inhibition of oxygen consumption and complex I and III activity, causing mitotoxicity and reduced ATP production. In fact, Sun et al. demonstrated in vitro that didanosine inhibited mitochondrial thymidine kinase 2 (TK2) and deoxyguanosine kinase (dGK), which are essential for mitochondrial function since they are involved in the initial rate-limiting phosphorylation of deoxynucleosides. Their inhibition led to mtDNA depletion, demonstrated by a 10% reduction in cytochrome c oxidase subunit II (COX II) protein synthesis, which is encoded by mitochondrial DNA [235].

### 3.9. Zonal Heterogeneity in Drug-Induced Lipid Accumulation

A difference in glucose and fatty acid metabolism has been observed in hepatocytes with respect to proximity to the portal triad, probably due to the existing gradient of nutrients and oxygen. While periportal hepatocytes are more involved in gluconeogenesis and β-oxidation of fatty acids, glycolysis and lipogenesis occur at a higher rate in centrilobular hepatocytes [236]. Additionally, lipids are distributed differentially within the liver lobule. One of the distinctive features of steatosis in adult NAFLD, in contrast to most pediatric NAFLD cases, is its predilection to start in zone 3 (centrilobular region); adult NASH presents steatosis in the form of ballooning, also typically localized in zone 3 [237]. An intralobular heterogeneous distribution of lipids has been observed for certain drugs. In particular, steatohepatitis has been observed in patients treated with agents such as methotrexate, amiodarone, perhexiline and tamoxifen. In these patients, NASH-like lesions were found, with centrilobular ballooning and perisinusoidal fibrosis [238,239]. In a study conducted on patients treated with methotrexate for rheumatoid arthritis, in 4.6% of patients with fatty liver, liver biopsy showed that the majority of them had a NASH-like pattern, with steatosis, centrilobular ballooning degeneration, and centrilobular perisinusoidal fibrosis [240]. However, it should be noted that the risk of significant fatty liver disease related to tamoxifen or methotrexate increases if the patient is already obese or diabetic, suggesting a synergistic effect [239]; so, the observed zonation in patients taking these drugs may be a consequence of the preexisting disease. Drug-induced microvesicular steatosis is restricted to a limited list of agents that primarily induce mitochondrial damage, including 2-arylproprionic acids, aspirin, tetracycline, valproic acid, fialuridine, antiretroviral drugs; for this kind of drug, heterogeneity in lipid deposition has not been reported [44].

## 4. Conclusions

Non-alcoholic fatty liver disease (NAFLD) and non-alcoholic steatohepatitis (NASH) today represent two of the major health challenges facing Western countries. A significant percentage of NAFLD and NASH is induced by the administration of drugs (DIFLD and DISH) for the treatment of extremely widespread pathologies such as hypertension and cardiovascular disorders, bacterial and viral infections, or tumors. A detailed understanding of the molecular mechanisms involved in the onset of NAFLD and NASH following the administration of specific drugs is certainly useful to minimize adverse effects in susceptible patients and to recommend alternative treatment solutions that reduce hepatotoxicity without interrupting the therapy.

## Figures and Tables

**Table 1:** List of the major drugs involved in steatosis onset and progression. They are classified according with their clinical use; their mechanisms of steatosis induction are also indicated.

Drug	Category	Mechanisms
Amiodarone	antiarrhythmic	Inhibition of OXPHOS, FAO, CPT1 Inhibition of MRC complex I and III Induction of ER stress Upregulation of SREBP-1c, ACLY, FAS, SCD1
Perhexiline	antiarrhythmic	Inhibition of OXPHOS, FAO, CPT1 Inhibition of MRC complex I and III
Diltiazem	antiarrhythmic	Conflicting data: observed steatosis with no described molecular mechanism
Verapamil	antiarrhythmic	Conflicting data: observed steatosis but also reduced inflammation, collagen deposition, lipid peroxidation, α-SMA and TGFβ1
Losartan	antihypertensive	Amelioration of NAFLD: reduced markers of hepatic fibrosis
Enalapril	antihypertensive	Macro- and microvesicular steatosis with increased inflammation
Nifedipine	antihypertensive	Conflicting data are reported: increased enzyme release, macro- and microsteatosis with fibrosis; upregulation of PPARγ receptor with consequent reduction in NASH, fibrosis and AST release
Tetracyclines	antibiotic	Inhibition of FAO (PPARα, CPT1), inhibition of lipid export, Upregulation of fatty acid transport, Upregulation of CYP2E1 and ROS production
Linezolid	antibiotic	Inhibition of mtDNA translation and OXPHOS activity
Rifampicin	antibiotic	Upregulation of de novo lipogenesis (SCD1, ACC, FAS), Upregulation of fatty acid uptake (PXR, PPARγ, CD36), Inhibition of FAO
Tamoxifen	antineoplastic	Inhibition of FAO through ERα/β receptors, Inhibition of mtDNA synthesis, Conflicting data: downregulation of FAS, but also induction of SREBP-1c
Toremifene	antineoplastic	Few cases of steatosis
Irinotecan	antineoplastic	Decrease in mtDNA synthesis, Inhibition of OXPHOS, Mitochondrial dysfunction, inflammation and fibrosis
5-Fluorouracil	antineoplastic	Increase in triglyceride accumulation, Mitochondrial dysfunction, Increase in peroxisomal beta-oxidation and ROS generation, Induction of JNK, IL-8 and ICAM.-1
L-Asparaginase	antineoplastic	Mitochondrial dysfunction, Alteration in VLDL secretion and metabolism,
Methotrexate	antineoplastic a	Decrease in OXPHOS, Downregulation of mitochondrial enzymes, Depletion of folate mitochondrial stores, Activation of stellate cell A2A receptor, leading to fibrosis
Valproic acid	antiepileptic	Acyl CoA sequestration and mitochondrial FAO inhibition, Reduction in citric acid cycle flux, Inhibition of OXPHOS, Inhibition of ATP production, Upregulation of CD36, Increase in oxidative stress and decrease in antioxidant defenses
Carbamazepine	antiepileptic	Decrease in microsomal cytochrome P-450 dependent enzyme activity
Dexamethasone	glucocorticoid	Hyperphagia (inhibition of leptin signaling pathway), Increase in ChREBP and SREBP-1c activity, Increase in de novo lipogenesis, upregulation of CD36, decreased secretion of VLDL, decreased FAO
Betamethasone	glucocorticoid	
Prednisolone	glucocorticoid	
Triamcinolone	glucocorticoid	
Salicylic acid (Aspirin)	NSAID	Conflicting data: mitochondrial dysfunction, increase in eNOS and decrease in iNOS and TNF-α, reduction in JNK activity
Acetaminophen	NSAID	Increase in ROS generation, Mitochondrial damage, GSH depletion, Accumulation of long-chain acylcarnitines, Reduction in PPARα expression, Alteration of FAO
Pirprofen	NSAID	Inhibition of FAO and natural CoA activity
Ibuprofen	NSAID	Inhibition of FXR transcriptional activity
Diclofenac	NSAID	Inhibition of mtFAO
Naproxen	NSAID	Inhibition of mtFAO
Ketoprofen	NSAID	Inhibition of mitochondrial function, ROS accumulation
Zidovudine (AZT)	NRTI	Inhibition of DNA polymerase γ, ER stress induction, Increase in SREBP-1c, Decrease in PPARα, phospho-AMP kinase and 3-keto-acyl-CoA thiolase, Inhibition of autophagy
Stavudine	NRTI	Inhibition of DNA polymerase γ, Inhibition of autophagy
Didanosine	NRTI	Inhibition of DNA polymerase γ, Inhibition of oxygen consumption and complex I and III activity

## Abbreviations

ACC	Acetyl CoA carboxylase
ACE	Angiotensin-converting enzyme
ACLY	ATP-citrate synthase
ACOX1	Acyl-CoA oxidase 1
AIDS	Acquired immune deficiency syndrome
ALP	Alkaline phosphatase
ALT	Alanine aminotransferase
ANGPTL4	Angiopoietin-like 4
Ang II	Angiontensin II
APAP	Acetaminophen
ApoB100	Apolipoprotein B100
AST	Aspartate transaminase
ATF4	Activating transcription factor 4
AZT	Zidovudine (3-azido-3′-deoxythymidine)
Bix	1-(3,4-dihydroxyphenyl)-2-thiocyanate-ethanone
BSO	L-buthionine-(S,R)-sulfoximine
CASH	Chemotherapy-associated steatohepatitis
CCl_4_	Carbon tetrachloride
CD36	Cluster of differentiation 36
CDAA	L-amino acid-defined
CHOP	CCAAT-enhancer-binding protein homologous protein
ChREBP	Carbohydrate regulatory element-binding protein
Cidea	Lipid droplet proteins cell death activator
Cidec	Cell death inducing DFFA like effector C
COX II	Cytochrome c oxidase subunit II
COX-2	Cyclooxygenase-2
CPT1	carnitine palmitoyltransferase 1
CYP2E1	Cytochrome P450 2E1
DEX	Dexamethasone
DGAT2	Diacylglycerol acyltransferase 2
dGK	Deoxyguanosine kinase
DIFLD	Drug-induced fatty liver disease
DIHS	Drug-induced hepatic steatosis
DILI	Drug-induced liver injury
DISH	Drug-induced steatohepatitis
DISH	Drug-induced steatohepatitis
DNL	De novo lipogenesis
DPD	Dihydropyrimidine dehydrogenase
eIF2α	Eukaryotic translation initiation factor-2α
ELP	Enalapril
eNOS	Endothelial nitric oxide synthase
ER	Endoplasmic reticulum
FAO	Fatty acid oxidation
FAS	Fatty acid synthase
FATP	Fatty acid transport protein
FOXO1	Forkhead box protein O1
5-FU	5-fluorouracil
FXR	Farnesoid X receptor
GC	Glucocorticoid
GR	Glucocorticoid receptor
GRP78	Glucose-regulated protein 78
GSH	Reduced glutathione
HAART	Highly active antiretroviral therapy
HADHβ	Beta-3-hydroxyacyl CoA dehydrogenase
HCV	Hepatitis C virus
Hepa1–6	Mouse hepatoma cells
HSC	Hepatic stellate cells
HIF-1α	Hypoxia-inducible factor 1 alpha
HFHC	High-fat, high calorie, high-fructose
HIV	Human immunodeficiency virus
HMOX1	Heme oxygenase 1
Sabi∆Hep	Inducible hepatocyte specific SAB deletion
iNOS	Inducible nitric oxide synthase
JNK	c-Jun N-terminal kinases
LPO	Lipid hydroperoxides
MAFLD	Metabolic associated fatty liver disease
MAPK	Mitogen-activated protein kinase
MCD	Methionine and choline deficient
MCR	Mitochondrial respiratory chain
MKP3	Mitogen-activated protein kinase phosphatase-3
mTOR	Mammalian target of rapamycin
MTP	Microsomal triglyceride transfer protein
MTTP	Microsomal triglyceride transfer protein
MTX	Methotrexate
NAFLD	Non-alcoholic fatty liver disease
NAPQI	N-acetyl-p-bemzoquinone imine
NASH	Non-alcoholic steatohepatitis
NLRP3	Nod-like receptor protein 3
NRTIs	Nucleoside reverse transcriptase inhibitors
NSAIDs	Nonsteroidal anti-inflammatory agents
OCA	Obeticholic acid
OXPHOS	Oxidative phosphorylation
PDGF-C	Platelet-derived growth factor-C
PEPCK	Phosphoenolpyruvate carboxykinase
PERK	Protein kinase-like endoplasmic reticulum kinase
PGC-1α	PPARγ coactivator 1α
PPAR	Peroxisome roliferator-activated receptor
RAAS	renin–angiotensin–aldosterone system
ROS	Reactive oxygen species
SAB	SH3BP5 protein
(GalNAc-Sab-ASO)	SAB N-acetylgalactosamine antisense oligonucleotide
SREBP-1c	Sterol regulatory element-binding protein 1C
sXBP1	spliced X-box binding protein 1
TAG	Triacylglycerol
TGFβ1	Transforming growth factor-β1
TGH	Triacylglycerol hydrolase
TK2	Thymidine kinase 2
TNF-α	Tumor necrosis factor alpha
TXNIP	Thioredoxin-interacting protein
VEGF	Vascular endothelial growth factor
VLDL	Very low density lipoprotein
VPA	Valproic acid
WT	Wild type
α-SMA	α-smooth muscle actin
γ-GT	Gamma-glutamyltransferase
